# Parasite Load Decrease during Application of a Safe and Easily Applied Antileishmanial Aminoglycoside Cream

**DOI:** 10.1371/journal.pntd.0002749

**Published:** 2014-05-22

**Authors:** Afif Ben Salah, Amor Zaâtour, Nathalie Ben Messaoud, Abdelhamid Kidar, Philip L. Smith, Karen M. Kopydlowski, Mara Kreishman-Deitrick, Carl J. Nielsen, Anne Novitt-Moreno, Janet H. Ransom, Gloria Morizot, Max Grogl, Pierre A. Buffet

**Affiliations:** 1 Institut Pasteur de Tunis, Tunis, Tunisia; 2 Regional Hospital of Gafsa, Gafsa, Tunisia; 3 United States Army Medical Materiel Development Activity, Fort Detrick, Maryland, United States of America; 4 Fast-Track Drugs & Biologics, LLC, North Potomac, Maryland, United States of America; 5 Assistance Publique - Hôpitaux de Paris, Paris, France; 6 Walter Reed Army Institute of Research, Silver Spring, Maryland, United States of America; University of Notre Dame, United States of America

## Introduction

Cutaneous leishmaniasis (CL) is a disfiguring illness caused by *Leishmania* species of protozoa, with over 350 million people at risk worldwide [1]. *Leishmania* parasites enter the skin through a sandfly bite, producing a papule or nodule that generally ulcerates [1]. Spontaneous resolution of CL ulcers may take months to years [2], and both active lesions and scars can engender stigma and cause disability [3].

There is no consensus regarding the optimum therapy for CL, and no single treatment approach fits all possible clinical presentations [1], [2]. However, because systemic treatments may produce considerable toxicity [1], [2], [4], localized therapy (e.g., intralesional antimonials, cryotherapy, thermotherapy, or intralesional injections plus cryotherapy) is now recommended in Old World CL (*L. major, L. tropica*) and in selected cases of New World CL [1], [2]. However, all present local therapy modalities have limitations, e.g., variable cure rates, pain, challenges and complexities associated with treatment administration (especially in children), and variable utility in patients with multiple lesions or lesions located on body areas where local treatment is impractical [1], [2], [5].

## Innovation

WR 279,396, which is a novel hydrophilic formulation of 15% paromomycin and 0.5% gentamicin, was developed at the Walter Reed Army Institute of Research as an efficacious, practical, and safe topical treatment for uncomplicated CL, in which the numbers and locations of CL lesions and the risk for disease dissemination are consistent with the use of a topical or other local treatment rather than a systemically acting drug. Paromomycin and gentamicin, two aminoglycoside antibiotics, inhibit protein synthesis in bacteria and accumulate in lysosomes where *Leishmania* multiply [5]. Paromomycin also has proven antileishmanial activity in vitro and is effective when administered parenterally for visceral leishmaniasis (VL) [1]. The inclusion of gentamicin, an aminoglycoside proven to attenuate *Leishmania* parasites [6], has increased the antileishmanial efficacy of paromomycin in rodents [5], [7], [8]. A novel hydrophilic vehicle employed in WR 279,396 is designed to aid drug penetration, and this new formulation overcomes deficiencies in efficacy and safety that had been described with previous topical paromomycin creams [2], [5], [7]. In two randomized controlled trials in Tunisia [5], [7], WR 279,396 was significantly more effective than placebo, with a 94% cure rate for the WR 279,396 group versus 71% in the placebo group (p = 0.0045) in a Phase 2 trial (n = 98) [5], and a 81% cure rate for the WR 279,396 group versus 58% in the vehicle control group (p = 0.0001) in a Phase 3 trial (n = 375) [7].

## Report of New Findings

A trial was conducted to determine whether the efficacy and safety of WR 279,396 would be impacted by the type of wound dressing employed (i.e., occlusive polyurethane dressing versus traditional gauze-and-tape) or by a simplified dosing schedule (i.e., once daily versus twice daily). Additional objectives were to evaluate the kinetics of parasite loads in lesions and to measure dermal aminoglycoside concentrations.

Sample size was determined as follows: The cure rate in the WR 279, 396 gauze-and-tape group was assumed to be 60%, and at least a 35% improvement was anticipated in the cure rate due to occlusion (polyurethane dressing). Based on this, for alpha  = 0.05 (one-sided) and beta  = 0.2, a total of 20 patients were required in each study group (i.e., total population n = 40). Once the study began, however, endpoint data could not be obtained for the first eight enrolled subjects because of a laboratory equipment failure in Tunisia. Hence, a protocol amendment was approved to recruit and randomize eight additional subjects, establishing a total population of 48. Therefore, a total of 48 Tunisian adults with parasitologically positive CL ulcers were randomized 1∶1 to receive WR 279,396 with either a polyurethane or gauze-and-tape dressing. Treatment was applied once daily for 20 consecutive days. No negative (placebo) control group was included in this study, the main objectives of which were to compare administration methodologies and to investigate parasite and aminoglycoside dynamics at the lesion level. In addition, the placebo cure rate (71%) had previously been determined in the earlier Phase 2 study [5] conducted in a similar population at the same study location.

A positive outcome (complete clinical response, CCR) was defined as a >50% improvement at day 50 follow by cure before day 90. Safety assessments included surveillance for local and systemic toxicity (i.e., audiometry, Romberg testing, blood chemistry). Outcome was assessed by an independent physician blinded as to treatment administered. *L*. *major* loads and paromomycin and gentamicin concentrations were determined in superficial and deep biopsy fragments obtained from lesions before and ten days after starting WR 279,396 applications. Parasite load was determined using a culture microtitration method [9], and results were compared by T-test between groups for the superficial and deep dermis at days 1 and 10. For aminoglycoside concentrations, biopsy samples were assayed for paromomycin and gentamicin isomer (C1, C1a, and C2) concentrations by liquid chromatography tandem mass spectroscopy. Since this study was the first to assess parasite dynamics in CL lesions being treated locally, the day 10 repeat biopsy timepoint was chosen to capture information midway in the 20-day treatment period, consistent with a near complete elimination of parasites by day 10 in animal models [8]. Also, from a clinical standpoint, it was reasoned that the CL ulcer would be more cosmetically amenable to biopsy at day 10, when the biopsy site could be incorporated into a lesion that was likely to be still open, rather than on day 20, when it might have already healed.

**Figure 1:**
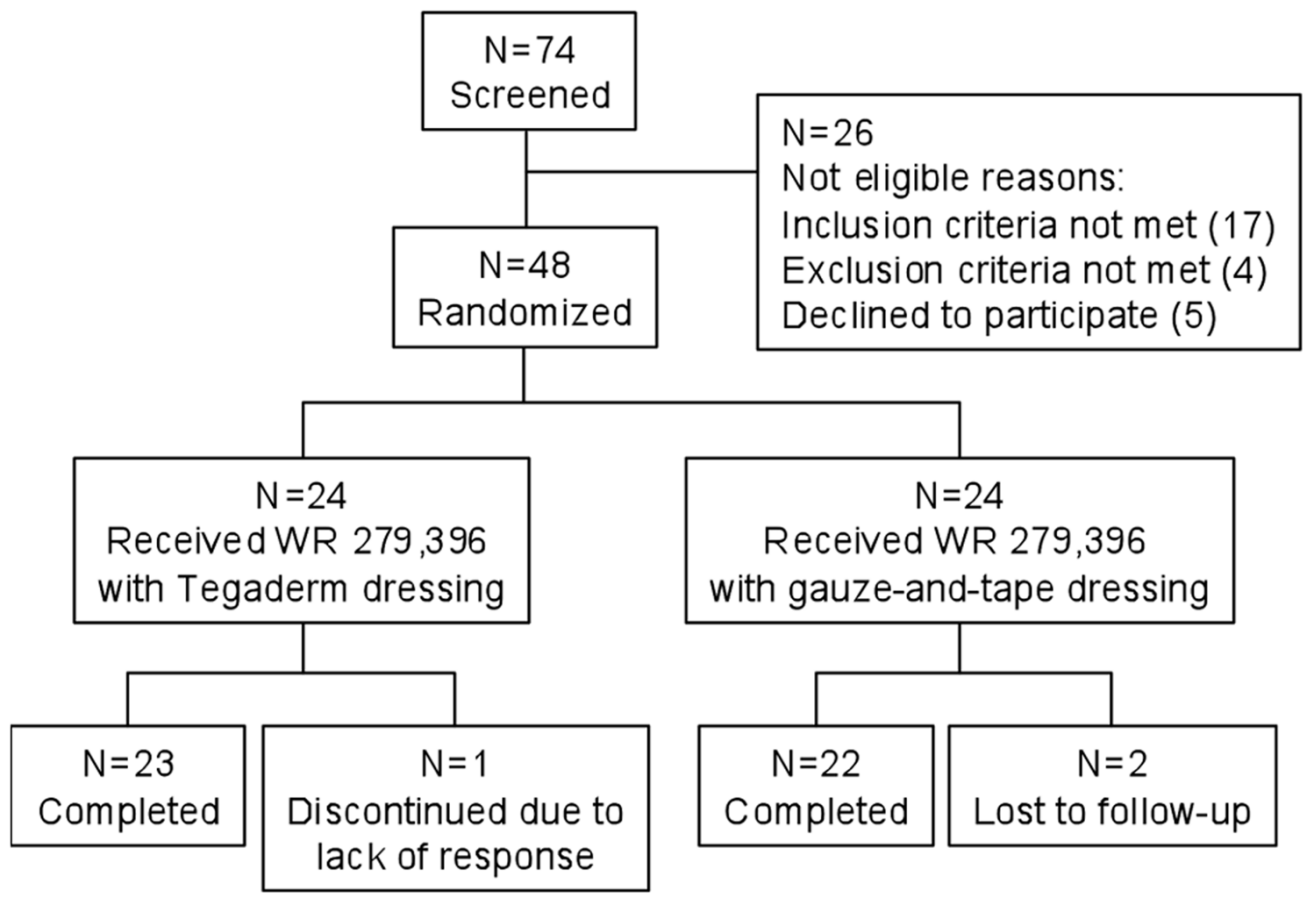
Summary of subject enrollment, treatment, and follow-up. Values are numbers of study subjects. Of the 74 subjects screened, 26 failed screening, most commonly due to having only one lesion that was already substantially healed (17 subjects). Forty-eight subjects were randomized, 24 in each study group. In the polyurethane group, one subject was discontinued due to an unsatisfactory result (enlargement of the index lesion at day 20). In the gauze-and-tape group, two subjects failed to return to the study site and were lost to follow-up. Forty-seven of 48 randomized subjects received a full 20-day course of WR 279,396 as planned in the protocol; one subject in the polyurethane group failed to return for further treatment after day 6.

Compliance was excellent (Figure 1) in both study groups (Table 1), with 97.9% of subjects completing their full 20-day treatment course. In the ITT population (all randomized subjects), the CCR rates were not statistically different between the two study groups (gauze-and-tape 91.7% versus polyurethane 79.2%, p = .42), although there was a trend towards increased efficacy with the more universally available gauze-and-tape dressing (Table 2). Applications performed once a day in this study were associated with a CCR rate (91.7%, gauze-and-tape group; total combined CCR rate 41/48 subjects, 85.4%), which was comparable to the result obtained with twice-daily dosing (Table 2) in the previous Phase 2 trial (CCR rate 94%) [5].

**Table 1:** Baseline characteristics of study patients.

	Polyurethane (n = 24)	Gauze-and-Tape (n = 24)
**Gender – n (%)**		
Male	12 (50.0)	11 (45.8)
Female	12 (50.0)	13 (54.2)
**Age (years)**		
Mean	39.0 (22.4)	35.9 (16.5)
Median	38.5	37.5
Range	(15,74)	(15,70)
**Baseline Medical History – n (%)**		
Diabetes	4 (16.7)	2 (8.3)
Allergies, including skin	2 (8.3)	3 (12.5)
**Index Lesion Character – n (%)**		
Located on Extremity	23 (98.5)	24 (100)
Located on Leg	15 (62.5)	19 (79.2)
Duration ≤2.5 months	19 (79.2)	19 (79.2)
**Index Lesion Area (Ulceration)**		
Mean (SD) – mm^2^	127.07 (147.39)	157.69 (179.52)
Median – mm^2^	73.32	72.93
Range – mm^2^	(15.6, 651.8)	(8.8, 599.4)
**Index Lesion Area (Induration)**		
Mean (SD) – mm^2^	360.77 (262.57)	454.33 (345.27)
Median – mm^2^	258.99	326.31
Range – mm^2^	(65.0, 1116.2)	(65.7, 1254.0)

**Table 2:** CCR rates.

Endpoint	Polyurethane	Gauze-and-Tape	p-value
CCR Rate† – current trial	19/24 (79.2%)	22/24 (91.7%)	.42*
CCR Rate – predecessor trial	47/50 (94.0%)	---	.10‡

† CCR rate defined as 100% re-epithelialization of the index lesion by day 50, or a >50% reepithelialization of the index lesion by day 50 followed by complete reepithelialization on or before day 90, with no relapse ever having occurred from day 50 through day 90.

*Fisher's exact test comparing the polyurethane group to the gauze-and-tape group.

‡ Fisher's exact test comparing the polyurethane group in the current trial to the polyurethane group in the predecessor trial.

Compared with baseline (Table 3), parasite loads at day 10 were reduced 13.2-fold and 52.8-fold in the superficial dermis (Table 4); and 55.0-fold and 76.8-fold in the deep dermis, in the polyurethane and gauze-and-tape groups, respectively. Concurrent with this reduction, potentially therapeutic levels of paromomycin were observed in biopsies of WR 279,396-treated lesions at day 10. Paromomycin levels (Table 5) ranged from 0.66 to 195.36 µg/g in the superficial dermis and from 0.73 to12.08 µg/g in the deep dermis at that time. In comparison, when paromomycin is administered intramuscularly for VL, peak plasma levels range from 18.3 µg/mL (±8.86) to 20.5 µg/mL (±7.01), while trough levels range from 1.31 µg/mL (±4.16) to 4.53 µg/mL (±6.71) [10]. Dermal paromomycin levels in the current study were also within the range of concentrations (0.554 to 2.4 µg/mL) shown to inhibit *L. major* amastigotes in vitro [11].

**Table 3:** Parasite loads in the superficial and deep dermis.

Parasite Load	Polyurethane Group	Gauze-and-Tape Group	t-test p-value between Groups
**Study Day 1, n**	19	17	
**Superficial dermis (10^6^/g)**			.15
Geometric mean	2,072	15,338	
Median	1,512	11,806	
Range (min, max)	(250, 1.1×10^5^)	(236, 2.1×10^6^)	
**Deep dermis (10^6^/g)**			.25
Geometric mean	2,496	10,301	
Median	1,543	8,000	
Range [min, max)	(236, 1.x10^5^)	(193, 7.9×10^5^)	
**Study Day 10, n**			
**Superficial dermis (10^6^/g)**			.09
Geometric mean	157	304	
Median	150	273	
Range (min, max)	(21, 2,540)	(20, 4,000)	
**p-value (paired t-test between days)**	<.0001	<.0001	
**Deep dermis (10^6^/g)**			.08
Geometric mean	45.4	145.0	
Median	62.0	157.0	
Range (min, max)	(11, 125)	(16, 4,000)	
**p-value (paired t-test between days)**	<.0001	<.0001	

**Table 4:** Parasite load fold-reduction ratios.

Parasite Load Fold-Reduction†	Polyurethane Group	Gauze-and-Tape Group	t-test p-value*
N	19	17	
**Superficial dermis (10^6^/g)**			.45
Geometric mean	13.2	52.8	
Median	11.1	58.8	
Range (min, max)	(0, 1,810)	(0, 3,176)	
**Deep dermis (10^6^/g)**			.35
Geometric mean	55.0	76.8	
Median	28.3	66.3	
Range (min, max)	(2, 4,655)	(8, 1,082)	

† The parasite load fold-reduction ratio is calculated as parasite load at day 1 divided by parasite load at day 10.

*Polyurethane group versus gauze-and-tape group.

**Table 5:** Geometric mean aminoglycoside concentrations in superficial and deep dermis.

Study Group	Geomean Paromomycin (µg/g)			Geomean Total Gentamicin (µg/g)		
	Superficial (n)†	Deep (n)	p*	Superficial (n)	Deep (n)	p
Polyurethane (n = 20)‡	10.84 (16)	3.54 (11)	.013	1.58 (10)	0.68 (3)	.004
Gauze-and-Tape (n = 18)	9.45 (13)	2.16 (8)	.033	2.48 (5)	0.26 (1)	.064

† Number of samples tested.

*Wilcoxon Rank Sum Test two-sided p-value for superficial versus deep dermis.

‡ Number of samples with detectable levels.

[Note: The lower limit of quantitation (LLOQ) was 20.0 ng/mL for paromomycin, 2.64 ng/mL for gentamicin C1, 2.45 ng/mL for gentamicin C1a, 4.25 ng/mL for gentamicin C2, and 50 ng/mL for total gentamicin.]

Consistent with its relatively low (0.5%) concentration in WR 279,396, gentamicin was detectable less frequently and at lower levels than paromomycin.

Possibly due to the low number of treatment failures, combined with a wide inter-individual variation in initial parasite loads and parasite load decrease at day10, no correlations were found between the reduction in parasite load at day 10 and various parameters of treatment outcome (e.g., CCR, time-to-healing, reduction in lesion size over time).

The treatment was well tolerated, causing only mild-to-moderate local reactions, primarily erythema (29.2% of subjects in the polyurethane group; 8.3% in the gauze-and-tape group) (Table 6).

**Table 6:** Summary of Adverse Events.

	Polyurethane Group (n = 24)	Gauze-and-Tape Group (n = 24)	Total Population (n = 48)
**Number of Reported Adverse Events**			
Total Adverse Events†	30	18	48
Mild	27	15	42
Moderate	3	3	6
Severe	0	0	0
**Subjects with Any Adverse Event – n (%)**	16 (66.7)	7 (29.2)	23 (47.9)
**Subjects with Any Local Toxicity** * **– n (%)**	9 (37.5)	4 (16.7)	13 (27.1)
Erythema‡	7 (29.2)	2 (8.3)	9 (18.8)
Edema‡	2 (8.3)	3 (12.5)	5 (10.4)
Pain	1 (4.2)	3 (12.5)	4 (8.3)
Subjects with concurrent localized infection 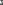 – n (%)	1 (4.2)	3 (12.5)	4 (8.3)
Subjects with concurrent polyurethane allergy – n (%)	4 (16.7)	---	4 (8.3)
**Subjects Reporting Vestibuloauditory Symptoms – n (%)**			
Decreased hearing	0 (0.0)	0 (0.0)	0 (0.0)
Tinnitus	0 (0.0)	0 (0.0)	0 (0.0)
Vertigo	2 (8.3)§	0 (0.0)	2 (4.2)
**Subjects with Evidence of Systemic Toxicity – n (%)**			
Abnormal audiometry	0 (0.0)	0 (0.0)	0 (0.0)
Abnormal Romberg	0 (0.0)	0 (0.0)	0 (0.0)
Abnormal serum creatinine 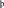	2 (8.3%)	0 (0.0)	0 (0.0)

† Total adverse events encompasses all intensities. Mild  =  no interference with daily activities; moderate  =  interferes with daily activities; severe  =  daily activities interrupted.

*Subjects with multiple symptoms were counted in all applicable symptom categories.

‡ In skin surrounding the index lesion.

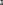
Localized infections generally occurred at the biopsy site, although a causal relationship to the biopsy was not established.

§ One subject suffered from pre-existing anemia and cardiac arrhythmia.

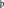
Mild, clinically insignificant elevations in serum creatinine were noted in both subjects at day 10 and resolved by day 20.

## Application

WR 279,396 is being developed to exploit the antileishmanial activity of its component aminoglycosides, while affording good tolerability and ease of administration. As in two other trials of WR 279,396 against *L. major* CL [5], [7], this study showed that cure rates in WR 279,396-treated patients were in the 80%–90% range, with most patients cured by day 50. In addition, a statistically significant, 13.2-fold to 76.8-fold reduction in parasite load occurred at day 10 in both study groups, concurrent with potentially therapeutic levels of paromomycin in WR 279,396-treated lesions. This study represented the first time in which the concentration of culturable parasites was measured in CL lesions being treated locally. Over ten days, parasite loads decreased equally well in the superficial and deep dermis, and by the same order of magnitude that had been observed in the spleens of patients with visceral leishmaniasis treated with systemic antimony [12].

In this study, the toxicity of WR 279,396 was limited to brief, mild-to-moderate local reactions that occurred in fewer than 30% of patients. Furthermore, WR 279,396 treatment was easy to apply under field conditions. These findings represent true therapeutic advantages in light of the potential toxicity associated with parental treatments and the drawbacks of alternative localized CL treatments: second degree burns and secondary infections with thermotherapy, treatment expense and need for specialized clinician training with cryotherapy, and significant local pain with intralesional antimonial injections [1], [2].

Thus, three independent comparative trials have now established the convincing efficacy of WR 279,396 against CL. This topical therapy is easy to apply and far less painful and less toxic than reference interventions (intralesional injections, systemic antimony).WR 279,396 represents an innovative and patient-friendly treatment approach for affected residents in CL endemic areas—especially children—as well as for travelers (see Box 1 for a summary of key characteristics of WR 279,396).

**Box 1. Advantages and Disadvantages of WR 279,396 Topical Antileishmanial Treatment**

**Advantages:**

1. WR 279,396 is clinically effective, showing cure rates in the 80%–90% range with a concurrent substantial reduction (up to 76.8-fold in ten days) in dermal parasite load.
2. WR 279,396 is well tolerated. Since it causes no systemic toxicity, WR 279,396 can be administered without the need for clinical laboratory testing/monitoring. Local toxicity, primarily erythema, occurs in fewer than 30% of subjects.
3. WR 279,396 is well adapted for wide use in endemic countries where many patients are children. It is easy to apply, especially when compared with intralesional injections and parenteral therapies. Also, it can be administered by health professionals with a minimum of training in medically underserved CL-endemic areas.

**Disadvantages:**

1. Topical treatments such as WR 279,396 may not be appropriate for disseminated forms of CL, CL lesions in the few areas of the body where a topical drug is impractical, or in cases of concomitant mucosal involvement.
2. WR 279,396 may not be effective against all *Leishmania* species. It has been tested primarily in areas endemic for *L. major*, and is currently being evaluated in an *L. panamensis*-endemic region and in travelers.
3. WR 279,396 is not yet widely available.

## Supporting Information

Checklist S1 **CONSORT checklist.**
(DOC)

Protocol S1 **Study protocol.** Protocol version dated 14 March 2010.
(PDF)
