# Supplementary material for: Parasite Load Decrease during Application of a Safe and Easily Applied Antileishmanial Aminoglycoside Cream
Source: PLoS Negl Trop Dis. 2014 May 22;8(5):e2749. doi: 10.1371/journal.pntd.0002749 (PMC4031053; doi:10.1371/journal.pntd.0002749)
Supplement: Protocol S1 — Study protocol. Protocol version dated 14 March 2010. (PDF) [file pntd.0002749.s002.pdf]

Topical Treatment of Old World Cutaneous Leishmaniasis with WR 279,396 (paromomycin/gentamicin ointment): Efficacy and tolerance of a regimen using an occlusive polyurethane dressing

PROTOCOL No. A-9768.2

Version 9

14 March 2010

1.0 GENERAL INFORMATION

IND/Date of submission	50,098/
CPP Protocol #/Date Approved	No. _____ / (mm/dd/yy)
Sponsor	Department of Defense (DOD)<br>Office of The Surgeon General of the Army
Sponsor Representative:	Commander<br>U.S. Army Medical Materiel Development Activity (USAMMDA)<br>622 Neiman Street<br>Fort Detrick, Maryland 21702-5012<br>(301) 619-7645/7643<br>Jerry.pierson@amedd.army.mil
Principal Investigator :<br>Afif Ben Salah, MD, PhD	Institut Pasteur, Tunisia<br>Phone 011-216-71-792-429 Fax: 011-216-71-791-833
Subinvestigators:<br>Nathalie Messaoud, MD	Institute Pasteur, Tunisia<br>Phone 011-216-71-792-429 Fax: 011-216-71-791-833
Scientific Expert and Consultant:<br>Pierre Buffet, MD, PhD	Pôle de Recherche Biomédicale, Centre Médical de l'Institut Pasteur, Institut Pasteur,<br>25-28 rue du Dr. Roux, 75015 Paris, France<br>Phone: 33-(0)1- 44389187 Fax: 33-(0)1-40613019
Study Coordinator and Consultant:<br>Max Grögl, PhD	Tripler Army Medical Center<br>Department of Clinical Investigation<br>Honolulu, Hawaii<br>Phone: 00 180 84337171
Coordinator and logistical support<br>Gloria Morizot, MD	Pôle de Recherche Biomédicale du Centre Médical de l'Institut Pasteur, Institut Pasteur,<br>25-28 rue du Dr. Roux, 75015 Paris, France<br>Phone: 011-33-(0)1-40613817 Fax: 33-(0)1-40613019
Monitor:<br><br><br><br><br>Shirley Roach, R.N.	USAMMDA<br>Ft. Detrick, MD<br>Phone : 001-301-619-6828
Medical Monitor<br>Nissaf Ben Alaya, MD	Institut Pasteur de Tunis<br>13 Place Pasteur BP, 74 Belvedere 1002, Tunis, Tunisia<br>Phone: 011- 216 71 783 022

Study Location:	Tunis, Tunisia
Institute Pasteur, Tunis, Tunisia	Institut Pasteur de Tunis<br>13 Place Pasteur BP, 74 Belvedere 1002, Tunis, Tunisia
Study Duration:	17 Months
Anticipated Start Up Date:	November 2005
Required No. of Volunteers:	40

TABLE OF CONTENTS

1.0 GENERAL INFORMATION **2**

2.0 BACKGROUND **6**

3.0 STUDY SUMMARY **6**

4.0 STUDY OBJECTIVES **7**

5.0 FACILITIES TO BE USED **8**

6.0 BACKGROUND OF LEISHMANIASIS **8**

6.1 Natural history and drug treatment of Old World cutaneous leishmaniasis 8
6.1.1 Old World cutaneous leishmaniasis 8
6.1.2 Cutaneous leishmaniasis in Tunisia 9

6.2 Present topical agents 10

6.3 WR 279,396 Preclinical Efficacy 11

6.4 Clinical Efficacy of WR 279,396 12
6.4.1 Summary of Phase 2 studies 12

6.5 Toxicity of WR 279,396 13
6.5.1 Preclinical toxicity of WR 279,396 13
6.5.2 Clinical toxicity of components of WR 279,396 13
6.5.4 Clinical toxicity of WR 279,396 17
6.5.5 Summary of toxicity 17
6.5.6 Chemistry and manufacturing 18

7.0 SUMMARY AND RATIONALE FOR THE PILOT PROOF OF CONCEPT PHASE 2 STUDY: 20 DAYS OF TREATMENTSTUDY WITH/WITHOUT OCCLUSION **18**

8.0 EXPERIMENTAL PLAN **19**

8.1 Trial Design 19

8.2 Trial endpoints 20

8.3 Study Population/Site 20

8.4 Number and randomization of lesions 21

8.5 Inclusion criteria 21

8.6 Procedures to evaluate inclusion/exclusion criteria 22

8.7 Exclusion (Non-inclusion) criteria 22

8.8 Duration of Study 23

8.9 Dosages and Administration of Drugs 23

8.10 Determination of adverse reactions 23
8.10.1 Determination of side effects 24
8.10.2 Determination of laboratory evidence of side effects 24

8.11 Establishment of diagnosis 24

8.12 Definition of responses using clinical criteria 25

8.13 Definition of cure ... 25
8.14 Criteria for Early Withdrawal from the Study ... 25
8.15 Concomitant medications ... 26
8.16 Departure from the protocol ... 26
8.17 Procedural timetable ... 27

9.0 SAMPLE SIZE AND DATA ANALYSIS ... 28
9.1 Sample size calculation ... 28
9.2 Efficacy calculations ... 28
9.3 Treatment randomization codes ... 28
9.4 Missing or unused data ... 29

10.0 DIRECT ACCESS TO SOURCE DATA/DOCUMENTS ... 29
11.0 QUALITY CONTROL AND QUALITY ASSURANCE ... 29
12.0 ETHICS ... 29
13.0 DATA HANDLING AND RECORDKEEPING ... 30
14.0 FINANCING ... 30
15.0 PUBLICATION POLICY ... 30
16.0 VOLUNTEER REGISTRY DATA BASE REQUIREMENTS ... 30
17.0 ASSESSMENT OF SAFETY ... 31
17.1 Safety Parameters / Adverse Events ... 31
17.1.1 Adverse Event ... 31
17.1.2 Serious Adverse Event ... 31
17.2 Recording and Reporting Adverse Events ... 32
17.2.1 Documenting Adverse Events ... 32
17.2.2 Reporting Adverse Events ... 32
17.2.3 Follow-Up of Adverse Events ... 33
17.3 Study Specific Definitions and Exceptions ... 33

18.0 ROLES AND RESPONSIBILITIES OF THE STUDY PERSONEL ... 33
19.0 REFERENCES ... 34
20.0 SIGNATURE OF INVESTIGATORS AND MEDICAL MONITOR ... 37

Appendix A (Adverse or Unexpected Event Determination – Side Effects and Grades)

2.0 BACKGROUND

An analysis of the data from the recent WR 279,396 study (HSRRB Log # A-9768.1) indicates that by day 50, cure rate was 91.8% in the treatment group and 73.2% in controls (x^2 = 5.60, p= 0.017) and complete cure response was 95.8% among the former versus 75% among the latter (x^2 = 8.04, p= 0.004). The global healing rate is already much higher than the previous (placebo) 25% healing rate in the same area using the same evaluation criteria (Ben Salah 95). The occlusive dressing used during the ongoing trial may have positively influenced the healing rate, by enhancing drug diffusion into the deep dermis and accelerating the reepithelialization process. The practicability of polyurethane adhesive dressings was not perfect however. Local reactions (mild pain, redness and edema) was noticed among 22 patients (11 among the treatment group (22.4 %) and 11 among controls (28.6 %), NS. Although no patient stopped drug applications, topical treatment might be improved by simplifying the regimen. WR 279,396 without occlusion was superior to placebo in a Colombian Phase 2 study (Soto 02) making it the reference schedule. No tolerance problems were observed in this trial.

The Office of the Surgeon General, United Sates Army represented by USAMMDA holds the IND for WR 279,396 and will continue the development of the product through a commercial pharmaceutical partner in order to obtain licensure by the FDA.

Thus, this Phase 2 study is to determine whether WR 279,396 with occlusion (a polyurethane dressing) is more effective than WR 279,396 without occlusion. Furthermore, a consistent difference in efficacy will be necessary to justify the relatively lower tolerance and higher cost of an occlusive dressing, and this study will help determine which form of dressing should be used.

Extensive objective and subjective local tolerance data will also be captured during this trial, as well as surrogate markers (parasite loads and aminoglycosides concentration in the deep dermis) that may help to determine the optimal number and duration of treatments. Finally, this study, which will be conducted from October 2005 through March 2007, will bridge the time gap between the 2004 Phase 2 study and the planned 2006 Phase 3 study. This will serve to keep the clinical team in Tunisia active and ready to start the next trial should that study be necessary.

There will be few changes to the ongoing IRB approved protocol. The study team in Tunisia and treatment schedule (daily application for 20 days) will be essentially the same, and the same facilities will be used.

3.0 STUDY SUMMARY

Fortyeight patients (48) with Old World cutaneous leishmaniasis will be randomly allocated to WR 279,396 treatment once a day for 20 days with an optimized polyurethane dressing (occlusion) (24 patients), or without occlusion (24 patients). All

patients will be rescued with the standard of care accepted in Tunisia, if the patient is not cured. The active ingredients of WR 279,396 are two aminoglycosides – paromomycin sulphate (15%) and gentamicin sulphate (0.5%) – in a base (AQIC).

Efficacy will be evaluated in terms of the number of lesions cured at D50 (D1 is the first day of treatment) with no relapse during 3 months post-treatment follow-up. Cure is defined as 100% reepitheliazation without relapse by 3 months.

Tolerance will be evaluated by local adverse reactions and by laboratory signs of systemic events.

In addition to the clinical evaluation of the CL lesions, the following parameters/clinical healing surrogates will be investigated:
(a) parasite load will be determined in superficial and deep lesional dermis samples at D0 and D10. The mean parasite reduction ratio (parasite load at D10/parasite load at D0) in each group will be compared;
(b) aminoglycoside concentrations (paromomycin and gentmicin) in superficial and deep infiltrated dermis in each group will be compared

One 4mm punch biopsy of the lesion randomized as explained above will be performed under epicutaneous plus transcutaneous anesthesia at day 0 and one 4 mm punch biopsy at day 10. The biopsy specimen will be divided in two: superficial dermis and deep dermis.

The results from this study will help determine the most practical treatment schedule and will answer questions that are crucial to improve the present treatment regimen with WR 279,396 which is twice a day for 20 days. This will facilitate the management of patients in the field and will help answer FDA questions during the licensing phase of the product.

4.0 STUDY OBJECTIVES

This study is designed to answer two primary questions: (a) is occlusion necessary and useful, and (b) would administration of WR 279,396 once-a-day for 20 days be as effective as twice a day for 20 days when compared to the results from study A-9768.1. The answers to these questions will help define the design of a pivotal Phase 3 study to support regulatory approval of this drug.

This study will evaluate the efficacy and tolerance of 20 days topical WR 279,396 once a day with occlusion in the treatment of Old World Cutaneous leishmaniasis. The reference schedule, i.e., 20 days topical WR 279,396 without occlusion, will be the randomized positive control group. No negative (placebo) control group will be included since the placebo cure rate has just been determined.

Decreasing the number of applications of the test drug (from 2x per day in A-9768.1) to 1x per day (this study) will likely decrease the number of times that dressing must be applied: This is expected to reduce the occurrence of AEs attributable to removal of the Tegaderm occlusive bandage. In addition, any new treatment regimen in cutaneous leishmaniasis should be amenable to local health system constraints, so our intent is to develop the simplest and shortest treatment schedule that will support easily application to all patients, especially for those in rural remote areas.

5.0 FACILITIES TO BE USED

Patients with presumed leishmaniasis would be screened in the endemic area of Sidi Bouzid and El Mnara and examined and enrolled in the study by a team from the Institut Pasteur in Tunisia. Patients will be treated as outpatients. This area has a laboratory that routinely performs standard clinical tests (urinalysis, serum chemistries, complete blood counts). All the parasitological exams will be done at the Clinical Laboratory of the regional hospital of Sidi-Bouzid in Tunisia.

6.0 BACKGROUND OF LEISHMANIASIS

6.1 Natural history and drug treatment of Old World cutaneous leishmaniasis

6.1.1 Old World cutaneous leishmaniasis

Cutaneous leishmaniasis typically presents as a papule that enlarges over approximately 1 month into an ulcer with raised edges and a necrotic center (1). The mean area per ulcer in one study was 400 mm2 (2). Nevertheless, rare ulcers are 2,000 $mm^{2,}$ and a rare patient may have 20 ulcers. The spontaneous cure rate over 6-8 weeks for cutaneous leishmaniasis caused by members of the L. major or L. tropica complex in the Old World ranges from 15-45% (3,4). The most reliable figures come from L. major disease in Tunisia and Iran, for which rates of 20-44% are reported (3,4). Although frequently follow-up in such studies is performed for 1 year, in a recent study of 27 relapsed lesions (5), only 2 occurred between 6 and 12 months after the end of therapy, and follow up for 6 months therefore seems sufficient.

Standard treatment is with intralesional injections of pentavalent antimonials. When 1-3 ml of Glucantime were administered intradermally once a week for 5 consecutive weeks to 20 patients with L. tropica in Syria, a cure rate of 76% was achieved (6). This regimen of Glucantime is considered successful in most L. tropica or L. major endemic areas, although these results are substantiated by only one controlled study. However, the regimen requires at least five painful intradermal injections which can hardly be performed in children and/or when lesions are located near eyes, mouth or nose. The overall cost of one intradermal injection is about $30 in France; $150 for a complete treatment course. For multiple and/or periorificial lesions that can't be injected, standard treatment is with pentavalent antimonials intramuscular at a dosage of 10-20 mg antimony (Sb)/kg/day for

10-20 days. This regimen of Pentostam or Glucantime is successful in Central and South America with a ~90% cure rate with 1 year follow up. A lower cure rate (50%) of cutaneous leishmaniasis was achieved in Algeria in a L. major endemic area (7). The regimen requires 20 days of injections for an outpatient dermatological disease, is expensive (~US$200 per course), and is potentially or frankly toxic to the heart, liver, pancreas, hematopoietic system, and musculoskeletal system (8).

The attempt to find alternatives to the above antimonial regimen has included oral agents such as ketoconazole, fluconazole and allopurinol, and short courses of antimonials and pentamidine. The oral agents have not been shown to be >75% curative (8, 35). On the other hand, a 10 day course of Pentostam, 20 mg/kg/day intravenously for 10 days (9), and a 4-injection course of pentamidine administered over 7 days (3 mg/kg qod for 4 injections)(9) have been reported to be ~90% curative in the New World.

6.1.2 Cutaneous leishmaniasis in Tunisia

Incidence: Three different forms of cutaneous leishmaniasis occur in Tunisia. *L. major* cutaneous leishmaniasis, by far the most frequent, is epidemic in Central and South of Tunisia, whereas L. infantum cutaneous leishmaniasis is found in the North and L. killicki in the Southeast. Over seventy percent of compulsory notified diseases in Tunisia are accounted for by zoonotic cutaneous leishmaniasis (ZCL), viral hepatitis, and tuberculosis. Therefore, ZCL is a major public health problem in Tunisia. Since 1982, an epidemic emerged in central Tunisia and expanded to the whole central and southern parts of the country (15/23 governorates are considered as endemic in 2002). The epidemics are cyclic and annually 2000 to 3000 cases are reported. The epidemic curve shows peaks and inter-epidemic periodicity of 5 to 6 years with an average incidence of ~20 per 100,000 persons. Prediction of epidemic peaks and geographic spread of disease is currently lacking which make prevention programs very difficult to design and implement. Key factors driving temporal-spatial trends of disease are presently unknown. These might include dynamics of rodent populations, dispersal of vectors, climate changes, vegetation and soil type, establishment of dense human settlements in areas where a sylvatic transmission of leishmaniasis is high (rodent-vector-rodent cycle).

Analysis of Tunisian surveillance system revealed that control measures were limited to case notification, passive detection and treatment of ZCL cases. Tunisian surveillance system is based on passive case detection of ZCL cases in the field at the primary health care centers. Transmission is seasonal and occurs during the spring and summer between April and May. ZCL cases emerge during the diapause season between October and April. Every month, during the season of cases emergence, all diagnosed cases at the primary health care centers are recorded in forms specially designed for this program and passed to epidemiology unit at the regional level (regional primary health care service of the governorate). Cumulative data is analysed at this level summarized and transmitted to the central level (Parasitic Disease Program manager at the primary health care direction located in the Ministry of Health [MoH] in Tunis). Analysis of ZCL data at the national level is performed there and displayed in the quarterly publication of the MoH. The age

distribution varies among the focuses. However, age distribution is skewed to young groups in the old foci and includes all age groups in the newly colonized ones.

Clinical characteristics: In a previous clinical trial, 75-80% of the lesions (all due to *L. major*) were at least partially ulcerative in nature and 17 to 20 % were either papules or nodules. Mean size of lesion at diagnosis was 23+/- 1.36 mm. Lesions were mainly located in uncovered parts of the body: legs: 41.4%, arms: 51.7%, trunk: 3.5% and face 3.5%. Few lesions had a rapid healing (6.9%) and 25.9% remained active until day 105. Direct smears originally positive at day 0 became negative among 56.4%, 71% and 92.3% in days 15, 45 and 105 respectively.

Epidemiology: The disease is now reemerging as an epidemic in rural areas in central Tunisia. We expect that patients will emerge from September to March with a peak in December. The medical team from Tunisia will screen approximately 10-15 patients at one time for entry into the study. Treatment and follow-up of the patients will be done on site (noted above). Members of the medical team will live in the study area until the end of the project.

6.2 Present topical agents

An alternative approach to a dermatological problem such as CL is a topical agent that can be applied directly to the lesion.

An Israeli topical formulation of paromomycin sulfate (15%) and methylbenzethonium chloride (12%) in soft white paraffin has been tried and is now marketed for Old World cutaneous leishmaniasis. Israeli L. major lesions treated with the formulation for 10 days cleared significantly more rapidly (100% cure at 21-30 days) than did untreated lesions on the same patients (100% cure at 51-60 days) (13). The primary agent in the topical formulation, paromomycin, is an aminoglycoside antibiotic analogous in structure to the antibacterial agent neomycin. Although neomycin is solely an antibacterial agent, paromomycin is clinically effective against protozoan and has good in vitro activity vs Leishmania amastigotes with an ED (100) of about 10 µg ml (14). The other agent in the topical formulation, methylbenzethonium chloride (MBCL), is a cationic quaternary ammonium antibacterial disinfectant present up to 0.02% concentration in shampoos that also have good in vitro antileishmanial activity [ED (100) = 5 µg ml] (15).

A different topical formulation of paromomycin sulfate (15%), 10 % urea in white soft paraffin was investigated in two randomized, double-blind, placebo controlled trials in children patients with L. major cutaneous leishmaniasis in Tunisia and Iran (3,4). The results were equivalent in both trials; the paromomycin ointment was safe, well tolerated and there was clear evidence of parasitologic efficacy. However, there were no clear clinical benefits with this formulation. The authors concluded that the ointment should be studied in longer or more frequent regimens in an effort to prevent parasitologic relapse and thus promote clinical improvement. In addition, these trials confirmed, that only

placebo-controlled clinical trials could demonstrate the efficacy of a new therapeutic agent in L. major CL since it is a fast healing condition.

In the New World, we determined the therapeutic index of paromomycin sulfate (15%) MBCL (5%) in paraffin against cutaneous leishmaniasis (16). A 10-day course, in combination with a 3-day course of Glucantime, was administered to Colombians with *L. panamensis* disease. Eight of 19 patients (42%) cured with 12 months follow up. Since this cure rate is low, it is clear that this topical formulation alone would not have been sufficiently curative. In a second cohort, a 10-day course of topical was administered with a 7-day course of Glucantime IM. Eighteen of 20 patients (90%) cured. Since 7 days of Glucantime by itself cures < 40% of patients, this trial indicates that 10 days of the topical plus 7 days of Glucantime, both of which are poorly curative by themselves, are highly curative in combination. Dermatological side effects consisted of burning and pruritus in 25% of patients and vesicle formation in 15% of patients. A second clinical study by Soto et. al., 1998 reconfirmed the first study.

These are the first reports that a regimen partially comprised of topical antimicrobials can be highly effective for New World cutaneous leishmaniasis. Nevertheless, the paromomycin/ MBCL/ paraffin topical formulation suffers in terms of efficacy (7 days of adjunctive injections were required for cure) and toxicity (40% of patients reported side effects).

6.3 WR 279,396 Preclinical Efficacy

Preclinical efficacy of WR 279,396 was compared to paromomycin sulfate (15%)/ MBCL (12%)/ paraffin in the topical treatment of cutaneous disease in BALB/c mice. Sixty-day old lesions were treated twice a day for 10 days, and the response to therapy was monitored over a further 70 days (TABLE). For ulcers due to L. major, >90% of lesions healed by day 20 after therapy in both treatment groups. Nevertheless, on day 10, 93 % of the lesions were healed with WR 279,396 while only 70% of the lesions were healed with the paromomycin sulfate (15%)/ MBCL (12%)/ paraffin formulation. For ulcers due to L. panamensis or *L. amazonensis*, all lesions treated with WR 279396 healed and did not relapse. Less than half of lesions treated with paromomycin-MBCL-Paraffin were healed by day 30, and all lesions had relapsed by day 70 after therapy. Since in contradistinction to paromomycin/MBCL, WR 279396 cured both Old and New World cutaneous leishmaniasis in mice, the latter formulation is the only topical suggested by preclinical efficacy data to be a candidate for the treatment of both New World and Old World cutaneous leishmaniasis.

TABLE: Efficacy of topical paromomycin-gentamicin-AQIC (WR 279,396 = WR) compared to paromomycin-MBCL-paraffin (PM) *in L. major, L. panamensis, and L. amazonensis* infected BALB/c mice.

Day After Therapy	*L. major* % Healed		*L. panamensis* % Healed		*L. amazonensis* % Healed	
	PM	WR	PM	WR	PM	WR
0	0	0	0	0	0	0
10	70	93	10	80	13	63
20	90	100	83	100	30	100
30	100	100	47	100	37	100
40	93	100	23	100	0	100
50	96	100	23	100	0	100
60	93	100	20	100	0	100
70	82	100	0	100	0	100

6.4 Clinical Efficacy of WR 279,396

6.4.1 Summary of Phase 2 studies

In 1999, a pilot phase 2 study was conducted in Colombia. This clinical study was a randomized, double blind trial of WR 279,396 compared to placebo (the cream base of WR 279,396), each administered twice-a-day for 20 days without occlusion.

In the active group in Colombia (in which WR 279,396 was applied without an occlusive dressing), 33 of 51 evaluable lesions cured (66%) at D50 and 5 lesions were non-evaluable (59% in intent-to-treat analysis). In the placebo group, 4 of 12 evaluable lesions cured (33%) at D50, and 5 lesions were non-evaluable.

There was no statistical difference between the pre-treatment sizes for cured vs failed lesions in WR 279396 patients (p = 0.7) nor in placebo patients (p = 0.8).

The 36 lesions that were treated with WR 279,396 were recognized as cured at the end of therapy on day 20 (20 lesions), at the first follow up on day 45 (13 lesions), and on day 90 (3 lesions). The 6 placebo lesions that cured were recognized on day 20 (1 lesion), day 45 (3 lesions), and day 90 (2 lesions). The mean cure time of 35 days for cured WR 279396 lesions was statistically different (p = 0.04) from the mean cure time of cured placebo lesions (56 days).

In 2003-2004, a Phase 2 study was conduced in Paris & Tunisia, Old World cutaneous leishmaniasis. 92 subjects were randomly assigned to treatment of which 90 subjects (80

from Tunisia and 10 from Paris) achieved all the follow-up period. Patients allocated treatment (n= 49) and placebo (n= 41) were similar for prognostic factors at inclusion (socio-demographic variables and clinical features of lesion). Each lesion was treated twice daily. Lesions were measured at the end of therapy, and at 50, 80 and 180 days after the institution of treatment. Cure rate at day 50 was 91.8% in the treatment group and 73.2% in controls (x^2 = 5.60, p= 0.017) and complete cure response was 95.8% among the former versus 75% among the latter (x^2 = 8.04, p= 0.004).

6.5 Toxicity of WR 279,396

6.5.1 Preclinical toxicity of WR 279,396

Dermatologic toxicity was evaluated in 3 GLP studies. In a study of allergic contact dermatitis (17), WR 279,396 was non-sensitizing. In a study of photo activated dermal irritation (18), WR 279,396 was non-irritative. In a study of photoallergic contact dermatitis (19) WR 279396 was concluded to have the potential to be a weak photoallergen.

Systemic toxicity after topical administration was evaluated in 1 GLP study (20). Rats (250 mg) were administered 10, 50, or 250 mg/kg of paromomycin in the form of WR 279,396 to abraded back twice a day for 5 days and then daily for 23 days. The change from twice daily dosing to once daily occurred on day 6 because by day 5, moderate to severe erythema (draize score of 3) was seen, primarily at the site of initial application of the material, on the mid and high dose animals. In contrast to twice-daily dosing, daily dosing did not result in clinical skin reaction. This indicates that irritation due to WR 279,396 in rats is rapidly (within 24 hrs) reversible. No significant abnormalities were seen on clinical pathology or histopathology, including BUN, creatinine, histopathology of the kidney, and histopathology of the sensory and vestibular hair cells of the ear, in the high dose group.

6.5.2 Clinical toxicity of components of WR 279,396

All chemotherapeutic agents in WR 279,396 (paromomycin and gentamicin) have been administered both topically and systemically to humans. Paromomycin was first isolated from a sample of Italian soil in 1959 (21), and clinical reports on its use date from the early 1960's. Gentamicin was released for general use in approximately 1968 (22). The target organs for both topical and systemic drug have therefore been identified.

6.5.2.1 Paromomycin

a) Systemic toxicity after parenteral injection

Paromomycin injectable is licensed in Italy with a recommended dosage of 500 mg twice-a-day intramuscularly (15 mg/kg for children aged 1-12 years) for 10 days (21: page 124).

Peak and trough serum levels with this regimen were 15-25 μg ml and 1-2 μg ml, respectively (21: page 88). Rat data suggested that nephrotoxicity and eighth nerve toxicity (acoustic > vestibular) may result from high dosing (21: pages 62-65). In addition, when 12 children were administered standard doses of 20-30 mg/kg/day for 15 days, there was no incidence of abnormal BUN, vestibular damage, or hearing damage (21: pages 95-96). When results from larger numbers of patients were summarized, systemic side effects in 2,397 patients were: renal hypofunction (2 patients), jaundice (1), ear buzzing (2), temporary cochlear damage (1), hypacusic (7) (21: page 101).

b) Systemic toxicity after oral administration

The only formulation of paromomycin presently marketed in the United States is oral paromomycin formulated as Humatin. The indication is intestinal amebiasis treated with 25-35 mg/kg daily for 5-10 days. Since "almost 100% of the drug is recovered in the stool", paromomycin is not absorbed through intact intestinal mucosa (23). It is therefore extremely unlikely that clinically significant absorption of paromomycin or gentamicin would occur through intact skin.

c) Topical toxicity after topical administration

In the study of paromomycin (15%)/MBCL (5%)/paraffin in Colombia, 8 of 20 patients (40%) experienced local symptoms (16). Three (3) patients experienced a burning sensation and pruritus during the first 3 days of topical administration and 2 patients experienced these symptoms intermittently throughout the 10 days of topical administration. Three (3) patients experienced the more severe local side effect of an eczematous reaction with erythema and vesicle formation. Two (2) of these three patients had vesicle formation only on the first 2 days of therapy, but one patient had vesicles throughout the 10-day period of treatment. In another study, 3 of 30 patients treated with paromomycin (15%) /MBCL (12%)/paraffin had to prematurely terminate therapy due to severe pain and edema whereas 0 of 9 patients treated with paromomycin (15%)/MBCL(5%)/paraffin reported such toxicity (24). This study suggests that side effects due to the formulation might be due to MBCL rather than to paromomycin. On the other hand, 2 of 20 patients administered paromomycin (15%)/ paraffin--a paromomycin-containing topical without MBCL-- experienced pruritus, tenderness, and edema (25).

6.5.2.2 Gentamicin

a) Systemic toxicity after parenteral injection

Gentamicin injectable is marketed in the USA with the customary recommended regimen of 1 mg/kg every 8 hours for 7-10 days (26). Peak serum levels with this regimen are approximately 5 μg/ml. Peak and trough levels beneath 12 μg/ml and 2 μg/ml are recommended to avoid systemic side effects, which primarily consist of nephrotoxity and neurotoxicity. It may be that appropriate peak levels correspond with minimization of ototoxicity and appropriate trough levels correspond with minimization of nephrotoxicity.

Nephrotoxicity is characteristically reversible. Among 71 patients given 77 courses of gentamicin, 32 demonstrated increases in BUN and serum creatinine probably due to gentamicin, and in all cases the indices of renal function returned to pretreatment values after the drug was stopped (22).

Neurotoxicity is manifested by eighth nerve dysfunction. Both vestibular damage (dizziness, vertigo) and auditory damage (high-toned tinnitus and hearing loss) are seen, but high-toned hearing loss is often the first sign of eighth nerve damage (26).

When 1,484 courses in which gentamicin was administered were examined, there was ototoxicity in 44 cases (3%) (27). Only vestibular dysfunction was seen in 2/3 of the cases; decreased vestibular and auditory function was seen in 1/6 of patients; decreased auditory function alone was seen in 1/6 of patients. All cases occurred less than 14 days after discontinuation of treatment. In 14 of 29 cases of vestibular dysfunction for which the duration of the abnormality was determined, the dysfunction was reversible. (28)

Data such as these have led to the following statements in the PDR: "The risk of toxic reactions is low in patients with normal renal function who do not receive Garamycin [gentamicin] injectable at higher doses or for longer periods of time than recommended." (22)

b) Systemic absorption and toxicity after topical administration

Stone et al (29) determined absorption of gentamicin from a 0.1% cream and a 0.1% ointment (i.e., gentamicin in bland petrolatum) applied to the skin for burn patients. Absorption was quantitated by bioassay on urine collected for 3 days after application of the topical formulation. When formulations were administered for 3 days, the maximal drug absorbance was 20% from the cream and 5% from the ointment. Absorption from the cream was highest for fresh wounds; absorption from the ointment was highest after the scar had separated. The authors postulate that when the burn had a large water content, as occurred in the immediate postburn period with local edema, absorption from the water-miscible cream was greatest. When there was a minimal amount of exudate after the scar separated, absorption from the ointment was maximal.

If a topical gentamicin is applied to a huge area of burned skin, absorption can be sufficient to result in typical gentamicin toxicity. Two patients with severe burns covering 60-80% of the body were treated solely with topical gentamicin, presumably the 0.1 % cream in use in 1974. Gentamicin levels in the serum reached 3.0 - 4.3 µg/ml, and both patients showed loss of hearing and of vestibular function (30).

Aminoglycosides have also been applied topically to the ear and to the eye. For Meniere's disease, intratympanic delivery of gentamicin is intended to expose the inner ear to sufficient drug to inhibit the excess activity of the vestibular apparatus that is causing vertigo. A standard protocol is to administer 1 ml of 27 mg TID (81 mg/day) for at most 4

days (31). For the intravitreal administration of aminoglycosides for endopthalmitis, the standard doses of 0.1-0.2 mg of gentamicin sulfate or 0.2-0.4 mg of amikacin sulfate may have the adverse effect of macular damage (32).
6.5.3 Clinical toxicity of WR 279,396: Phase 1 study (33)

The aims of the phase I study were to investigate irritancy and phototoxicity due to WR 279396. In addition, blood was drawn to determine systemic toxicity (absorption of the aminoglycosides, alteration of kidney function and auditory function). In the irritancy study, 30 volunteers were administered WR 279,396, placebo cream (the AQIC base that contained urea), and white petrolatum to separate areas on the upper arm in a double-blinded manner for 15 days over a 20 day period.

The results of the irritancy study are:

a) Active cream (WR 279,396): 12 volunteers had 1-4 erythematous papules for 1-6 of the 15 days of examined application; for 8 volunteers, the papules advanced to pustules; for 3 volunteers, the lesions evolved into erosions.

b) Placebo cream (AQIC containing urea): 29 volunteers had fine wrinkling of the skin for 1-8 of the 15 days of application; 26 had +/- erythema (faint redness) for 3-10 days; 9 had 1+ erythema (erythema and edema of entire site) for 1-2 days. No patient had 2+ or 3+ erythema (vesicular changes or bullous changes).

One volunteer had acute dermatitis that developed at the sites both of active and placebo cream, that resolved. The volunteer was rechallenged with all ingredients in WR 279,396 known to cause allergic contact dermatitis, with negative results.

The interpretation of the irritancy study is that WR 279,396 can produce follicular irritant dermatitis with erythematous papules, and less frequently pustules and erosions. In addition, the urea in the AQIC vehicle, when it decomposes and is unbuffered by the aminoglycosides in WR 279396, characteristically produces mild irritancy.

In the phototoxicity study, 10 of the volunteers also received a challenge with ultra-violet light to areas of skin to which either WR 279,396 or the control substance, white petrolatum, was previously applied for 30 minutes. WR 279,396 was no different from the control substance in the degree of erythema produced.

In terms of systemic toxicity: examination of blood drawn on the last day of application or the following day, revealed that no volunteer had measurable blood levels (> 100 ng/ml) of paromomycin or of gentamicin. There was no statistical change in a kidney function test (creatinine). In audiometric tests pre-and post- topical application, more volunteers showed increases in high-frequency hearing than showed decreases in hearing.

6.5.4 Clinical toxicity of WR 279,396

6.5.4.1 Summary of a Phase 2 study in the New World

In the 1999, randomized, double-blind pilot study of WR 279,396 compared to placebo, each administered twice-a-day for 20 days in Colombia, no patient demonstrated an increase in creatinine values to higher than normal values (1.5 mg/dL) by the end of therapy. In addition, side effects in both groups consisted entirely of grade 1 local pain (describe as pain that does not interfere with daily activity). This grade 1 pain lasted a mean of 4 days in 55% of active patients and 3 days in 33% of placebo patients.

In Colombian cutaneous leishmaniasis, WR 279,396 was therefore demonstrated to be a non-toxic topical formulation that significantly diminished lesion cure time.

6.5.4.2 Summary of a phase 2 study in the Old World

In 2003-2004, a phase 2 study was conduced in Paris & Tunisia to evaluate the efficacy of WR 279,396 as a treatment for, Old World cutaneous leishmaniasis. 92 subjects were randomly assigned to treatment of which 90 subjects achieved all the follow-up through the end of the study period. Patients were allocated treatment (n= 49) or placebo (n= 41) for 20 days twice-a-day with occlusion and followed for 6 months. Like the Colombian Phase 2 study, this study established the safety of WR 279,396. No systemic toxicity was found and only minor (grade 1) local toxicity was determined that resolved independently in 1-3 days without stopping treatment. WR 279,396 was safe, no systemic reactions were determined and no laboratory abnormalities were found. Only 22 patients (24.4%) had local reactions of short duration (1-3 days) (11 among the treatment group (22.4%) and 11 among controls (28.6%), NS) that required no treatment.

6.5.5 Summary of toxicity

The potential dermatological side effects of topical paromomycin consist of pruritus/ pain/ tenderness, erythema, edema, and vesicle formation. These side effects may result either from irritation or, particularly in persons previously sensitized to neomycin or gentamicin, allergic reactions.

Systemic side effects due to paromomycin and gentamicin are primarily due to kidney damage (increased BUN and creatinine) and 8th nerve damage (high-toned hearing loss, vertigo). At least for gentamicin, these systemic side effects generally occur when serum concentrations greater than that achieved with customary parenteral doses are administered. There are no studies in which the toxicity of paromomycin combined with gentamicin have been compared to the toxicity of the separate aminoglycosides. However, as mentioned above, in Colombia, WRAIR’s formulation show no signs of systemic side effects and prove to be safe.

In the phase 1 study of dermatological irritancy and photoirritancy, WR 279396 caused slight follicular irritancy in 40% of volunteers when administered for 15 days over a 20-day period of time.

6.5.6 Chemistry and manufacturing

The components are synthesized under GMP by Farmitalia (Paromomycin) and by Schering (Gentamicin). Formulation of WR 279,396 was performed under GMP by the University of Iowa. Analysis of formulated WR 279,396 was within 6% of labeled paromomycin amount and 10% of labeled gentamicin amount (April 2002).

Formulation stability was determined over 8 weeks. At 45^0C, there was a 48% decrement in paromomycin and a 36% decrement in gentamicin. At 35^0 C, there was a 17% decrement in paromomycin and a 9% decrement in gentamicin. At room temperature (19-26^0C), there was a 3% decrement in paromomycin and 2% decrement in gentamicin. Therefore, WR 279,396 must be stored at ~25^0 C or in the refrigerator (~5^0 C).

3MTegaderm is a product classified as a semipermeable adhesive BP and manufactured by 3M Health Care Ltd. 3M Tegaderm Absorbent Clear Acrylic Dressing is made of a unique construction that incorporates 3M Tegaderm Transparent Dressing as a top and a skin contact layer that sandwiches a proprietary acrylic polymer. Tegaderm is a sterile, waterproof, thin, transparent film dressing. It was designed to be applied over I.V. devices, clean skin, or wounds providing a breathable bacterial and viral barrier to outside contaminants. The dressing is available in a variety of sizes and shapes. 3M Tegaderm, which is permeable to both water vapor and oxygen, is impermeable to micro-organisms and once in position, it provides an effective barrier to external contamination, whilst producing a moist environment at the surface of the wound by reducing water vapor loss from the exposed tissue.

7.0 SUMMARY AND RATIONALE FOR THE PILOT PROOF OF CONCEPT PHASE 2 STUDY: 20 DAYS OF TREATMENTSTUDY WITH/WITHOUT OCCLUSION

A analysis of the data from the recent WR 279,396 study (HSRRB Log # A-97-68.1) indicates that by day 50, cure rate was 91.8% in the treatment group and 73.2% in controls (x^2 = 5.60, p= 0.017) and complete cure response was 95.8% among the former versus 75% among the latter (x^2 = 8.04, p= 0.004)

The Office of the Surgeon General, United States Army, represented by USAMMDA holds the IND for WR 279,396 (IND#50098) and will continue the development of the product through a commercial pharmaceutical partner with the goal of obtaining approval by the US FDA. If necessary, a Phase 3 study will be conducted with the new formulation of WR 279,396 in Paris and Tunis to start in October 2006.

Major questions remain to be addressed in order to accurately decide the appropriate arms of the pivotal Phase 3 study are: (a) is occlusion necessary and useful; and (b) would administration of WR 279,396 once-a-day for 20 days be as effective as twice a day for 20 days.
.

Any new treatment regimen in cutaneous leishmaniasis should be amenable to local health system constraints so our intent is to develop the simplest and shortest treatment schedule that will support easily application to children and soldiers in rural remote areas. A consistent difference of efficacy will be necessary to justify the relatively lower tolerance and higher cost of occlusive dressing. Decreasing the number of applications of the test drug will not only simplify the treatment but decrease the number of times that dressing is applied, thus diminishing the number of AEs triggered by peeling off the Tegaderm.

This study bridges the ongoing Phase 2 study that ended follow-up on November 2004 and the start of the Phase 3 trial.

8.0 EXPERIMENTAL PLAN

8.1 Trial Design

Fortyeight (48) patients of Old World cutaneous leishmaniasis will be randomly allocated to WR 279,396 treatment once-a-day for 20 days with occlusion (24 patients), without occlusion (24 patients). Patients not cured will be rescued with a conventional treatment accepted in Tunisia as determined by the primary care physician. The active ingredients of WR 279,396 are two aminoglycosides – paromomycin sulphate (15%) and gentamicin sulphate (0.5%) – in a base (AQIC).

Efficacy will be evaluated in terms of the number of lesions cured i.e. percentage of subjects cured at D50 (D1 is the first day of treatment) with no relapse during 3 months post-treatment follow-up. Cure is defined as 100% reepitheliazation without relapse by 3 months. Clinical improvement is defined as ≥50% but ≤100% reepitheliazation compared to pre-treatment lesion size at day 50 (± 7 days). Lack of clinical improvement at day 50 (± 7 days), relapse that is enlargement of the lesion compared to the previous measurement at any time after day 50 (± 7 days), or not demonstrating complete clinical response by 3 months will be a clinical failure. Time to cure will also be determined. Toxicity will be evaluated by local adverse reactions, and by laboratory signs of systemic events.

Induration of the lesion, as measured by the ball-pen technique, will be measured at the same time as lesion size. A photograph will be taken concurrently with these measurements to document the procedure and provide a visual record of the induration measurement and lesion size.

In addition to the clinical evaluation of the CL lesions, the following parameters/clinical healing surrogates will be investigated: (a) parasites loads will be determined in superficial and deep lesion dermis at D0 and D10. The mean parasite log-reduction ratio (log parasite load at D10/log parasite load at D0) in each group will be compared; (b) aminoglycoside concentrations in superficial and deep infiltrated dermis in each group will be compared.

One 4mm punch biopsy of the lesion randomized as explained above will be performed under epicutaneous plus transcutaneous anesthesia at day 0, and 4 mm punch biopsy at day10. The biopsy specimen will be divided in two: superficial dermis and deep dermis. The following battery of tests will be performed on each homogenized tissue: (i) parasite load by the culture microtitration technique and/or other kind of parasite load quantification (the same technique will be used throughout the study) (ii) in situ aminoglycoside concentration will be determined using liquid chromatographic/mass spectrometric/mass spectrometric assay. The biopsy will be conducted by a qualified health professional under the supervision of a Medical Doctor in the health center. Past history of bleeding and hygiene conditions will be carefully considered, such as sampling environment conditions, and availability of local anti-bleeding drugs.

The results from this study will help determine the most efficient treatment regimen for the upcoming pivotal Phase 3 study and will answer questions that are crucial to improve the present treatment regimen with WR 279,396 of twice a day for 20 days. This will facilitate the management of patients in the field and will help answer FDA questions during the licensing phase of the product.

Risk for volunteers will be minimal (two punch biopsies under local anesthesia, performed by experienced clinicians).

8.2 Trial endpoints

1. Primary endpoints: Lesion size and clinical response described as reepithelialization. Lesion size refers to ulcer size only, here, and the rest of the protocol.

2. Secondary endpoint: safety and tolerance of WR 279,396.

3. Third end point: D0 to D10 Deep Lesion Dermis Parasite Load Reduction Ratio.

8.3 Study Population/Site

The study population will be selected from adults (≥ 18y) patients and children above 15y in Tunisia (Old World). Direct benefits to all subjects enrolled in the study are: a) elimination of bacterial infections by keeping the lesion clean; b) direct access to a medical team that will perform a medical history, physical exam, dermatology exam, and

laboratory tests; c) complete parasitological diagnosis, d) the possibility of cure without parenteral or intralesional antimony treatment

The recruitment process will be based on active case detection of patients with typical lesions in the primary health care centers and schools of the study area in Sidi Bouzid and Elmnara. Suspected cases based on clinical and epidemiological criteria will be identified by the diagnostic team composed of nurses trained by the study team in Tunisia. The central team will review all suspected cases, verify clinical inclusion criteria and perform a direct exam and culture for parasites as usually practiced in the primary health care system. Patients with positive lesions will be asked for written informed consent after the study is explained in accordance with the protocol requirements. Those who agree to be included in the study will continue the whole process of laboratory and other clinical inclusion/exclusion tests. After performing all the tests as described in the protocol, the principal investigator will review all the information for every case and make the inclusion decision according to the protocol and the protocol checklist.

8.4 Number and randomization of lesions

Fortyeight lesions (48) on fortyeight patients (48) will be studied. Efficacy will be evaluated in terms of the number of lesions cured i.e. percentage of subjects cured at D50 (D1 is the first day of treatment) with no relapse during 3 months post-treatment follow-up. Cure is defined as 100% reepitheliazation without relapse by 3 months. If a patient has more than 1 lesion, all lesions will be treated with WR 279,396, but the results of only 1 lesion (primary lesion) will be used for parasitological evaluation, and kinetic/clinical surrogate analysis. The lesion chosen will be the uppermost, primary ulcerative, parasitologicaly positive lesion on the body that is in a biopsy friendly area, or if two lesions are equally uppermost, the patient’s left (located in the left half of the body) uppermost primary ulcerative lesion that is biopsy friendly. Biopsy friendly areas exclude the face, ears, fingers, toes and, in patients > 50 or with arterial or venous impairments, any area below the knees in patients older than 55 or with previous history of lower limbs venous or arterial insufficiency.

Patients will be enrolled in the study in the order they consent and given Subject’s Numbers.

8.5 Inclusion criteria

1. Age: ≥15 – ≤75 years old
2. Lesion character: each diameter (horizontal and vertical) of the lesion test must measure ≥ 7 mm, the lesion must be primarily ulcerative (i.e., not verrucous or nodular) and located in a biopsy friendly site of the body
3. Parasitological diagnosis: have cutaneous leishmaniasis proven parasitologically in lesion selected for inclusion in study (lesion test).
4. Informed consent: have given written informed consent to participate in the study: (i.e. patient or legal representative).

8.6 Procedures to evaluate inclusion/exclusion criteria

1. Medical history, with particular attention to skin, lymphatic system & mucosal membranes
2. Physical examination
3. Dermatological examination: including measurement of leishmaniasis lesions
4. Urinalysis
5. Complete blood count (WBC, platelets, Hgb)
6. Serum chemistries: creatinine, alanine aminotransferase (ALT) or aspartate aminotransferase (AST), glucose, Na, K.
7. Urine pregnancy test

8.7 Exclusion (Non-inclusion) criteria

1. Drug intolerance: history of known or suspected hypersensitivity or idiosyncratic reactions to aminoglycosides in the patient or immediate family members.
2. Previous use of antileishmanial drugs (within 2 months) or present use of routinely nephrotoxic or ototoxic drugs.
3. Potential for follow up: Have less than 4 months time remaining in present address and/or plans to leave the area for more than 30 days.
4. Extent of disease: More than 10 lesions or lesion ≥ 5 cm or a lesion less than 2 cm from eye, in the ear, or a lesion in the face, that in the opinion of the attending dermatologist could potentially cause significant disfigurement.
5. Location of disease: mucosal involvement.
6. Disseminated disease: clinically significant lymphadenitis with nodules that are painful and > 1 cm in size in the lymphatic drainage of the ulcer.
7. Concomitant medical problems: significant medical problems of the kidney or liver as determined by history and by the following laboratory studies:
   - Kidney: clinically significant abnormalities of urine analysis, serum levels of Creatinine, BUN, total proteins > upper limit of normal for the laboratory.
   - Liver: AST or ALT > upper limit of normal for the laboratory.
   - General: glucose, Na, K, > upper limit of normal or < lower limit of normal for the laboratory.

   Volunteers in whom these normal laboratory values are exceeded by less than 25% will not be automatically excluded. These volunteers will be evaluated on the basis of history, physical, as well as laboratory values.
8. Scheduled or ongoing pregnancy as determined clinical and biological criteria.
9. Presence of signs or symptoms of peripheral neuropathy, myasthenia gravis or neuromuscular block

8.8 Duration of Study

Each patient will be treated for 20 days, and be followed for 3 further months. Enrollment will start in October 2005 and the study will end in March 2007.

8.9 Dosages and Administration of Drugs

Test articles to be used are:

Group 1: WR 279,396 (paromomycin sulfate 15%, gentamicin sulfate 0.5%, water to dissolve the aminoglycosides, AQIC base) and permanently covered with a polyurethane dressing (Tegaderm). i.e., polyurethane-covered perforated gauze fixed to skin through hypoallergenic medical tape.
Group 2: WR 279,396 (paromomycin sulfate 15%, gentamicin sulfate 0.5%, water to dissolve the aminoglycosides, AQIC base) without the polyurethane dressing i.e., gauze fixed to skin through hypoallergenic medical tape.

The method of administration of topical in 2 groups will be as per SOP.

The topical preparations were prepared by Dr. Flanagan, University of Iowa, and distributed in 40-ml jars. The jars will be stored at 2–8°C at the Institut Pasteur in Tunis.

The method of administration of topical will be as per SOP. In brief, the lesion shall be appropriately disinfected. Then the formulation will be applied at a rate of about 0.0005 ml per 1 mm2 of lesion by covering uniformly the ulcer with a thin layer of ointment, once-a-day for 20 days. Once a day shall mean that the administration occurs 24 hours after the first administration (+/- 4 hr). Medical personnel shall record that the formulation was administered. For Group 1 lesion/medication will be permanently covered with a polyurethane dressing (Tegaderm) and undisturbed (not wiped off, not wetted) for 4 hr after each application.

Iso-enzyme analysis of the parasite isolated from the lesion will be completed after treatment has been started. If the iso-enzyme analysis is L. aethiopica, the volunteer will be referred to his primary health care provider for the appropriate standard treatment (probably Glucantime). Should the volunteer elect to receive another treatment, the volunteer will be dropped from the study at the start of glucantime or other antileishmanial drug. Since no *L. aethiopica* has been reported in Tunisia in the last decade, the likelihood of a volunteer being dropped for this reason is remote.

8.10 Determination of adverse reactions

8.10.1 Determination of side effects

On each day on which topical is administered, patients will be observed/questioned for the occurrence of the following local side effects (i) on the edge of lesion (ii) on surrounding healthy skin (Please see Case Report Form):

-pain by history	(none, mild, moderate, or severe)
-erythema	(none, mild=barely perceptible, moderate=well defined, severe=very red)
-edema	(none, mild=barely perceptible, moderate=well defined, severe=raised >2mm; life threatening = exfoliative)
-vertigo	(mild, moderate, or severe)
-tinnitus	(mild, moderate, or severe)
-diminished hearing	(mild, moderate, severe or profound)

Subjective evaluation

8.10.2 Determination of laboratory evidence of side effects

Blood will be drawn to re-determine creatinine on days 10 and 20.

Hearing and Romberg tests will be repeated on days 10 and 20 for monitoring the hearing function since the drug might be absorbed into the blood and can affect hearing. Of note, recent data from a cGCP Phase III study in India show that no clinically significant hearing and kidney AEs occurred in a cohort of > 400 patients (40.9% aged 5 – 15 years) treated with paromomycin IM 15 mg/kg for 21 days (Pr. Jha TK & Dr. Sinha PK oral communications, WorldLeish 3 Congress, Terrasini, Italy, 10 – 15 April 2005). The procedure for evaluating Hearing and conducting Romberg tests will be per SOP. In brief, (i) in the hearing test, the patient wears earphones that play tones of different frequency (pitch) and loudness into one ear or the other. The patient signals when he hears a tone by raising his hand. For each pitch, the test identifies the quietest tone the person can hear in each ear. The results are presented in comparison to what is considered normal hearing. (ii) Romberg test for evaluating vestibular function, by having the patient stand still with their heels together. The patient is asked to remain still and close their eyes. If the patient loses their balance, the test is positive.

8.11 Establishment of diagnosis

1. Each lesion to be evaluated for efficacy will be aspirated and/or scraped and/or biopsied.
2. Proof of infection will include the demonstration of motile promastigotes in aspirate cultures or microscopic identification of Leishmania amastigotes (by DifQuiK or Giemsa staining) in material obtained from lesions.

8.12 Definition of responses using clinical criteria

Lesions will be measured in two perpendicular directions, and photographs will be taken, prior to therapy, at the end of therapy, and at 50 days, 3 months, (D1 being the first day of treatment). Physically identifying features if present will be hidden in photographs. Patients will be identified by initials and not by name. Because the trial can't be blinded, clinical evaluation at D50 will be performed by an independent physician (Dr. Mourad Mokni) unaware of the occlusion process. Patients will be asked not to inform the evaluating physician if the lesion was covered.

Efficacy will be evaluated in terms of percentage of patients cured or improved at D50 (D1 is the first day of treatment) with no relapse during 3 months post-treatment follow-up.

1. Cure:	100% reepithelialization without relapse by 3 months.
2. Clinical improvement:	≥50% but ≤100% reepitheliazation compared to pre-treatment lesion size at day 50 (± 7 days).
3. Clinical failure:	a. Lack of clinical improvement at day 50 (± 7 days) after the beginning of treatment.<br>b. Relapse that is enlargement of the lesion compared to the previous measurement at any time after day 50 (± 7 days).<br>c. Not demonstrating complete clinical response by 3 months (i.e. not being cured by 3 months).

8.13 Definition of cure

Determination of cure will be based on clinical criteria. A lesion (ulcer) will be defined as cured if it has undergone a complete clinical response.

8.14 Criteria for Early Withdrawal from the Study

Any one of the following criteria will be considered sufficient to cause the withdrawal of the patient from the study.

1. Adverse effects (particularly those listed in section 7.10.2) reported by the patient or medical personnel that in the opinion of the principal investigator pose a serious risk to the patient.
2. Clinical failure or relapse. Patients who require re-treatment for failure or relapse will be removed from the protocol and treated with the standard of care, probably 0.5 – 3.0 ml of Glucantime intralesionaly once per week for a maximum of 5 weeks or in case of

multiple lesions (n≥4) Glucantime at 20 mg SbV/kg /day for a maximum of 20 days) or the attending physician drug of choice.

8.15 Concomitant medications

If concomitant medical problems occur, medications that are possibly effective against cutaneous leishmaniasis (antimony, amphotericin B, pentamidine, injectable paromomycin, fluconazole, ketoconazole, allopurinol) will only be used as a measure of last resort and this patient will be withdrawn from the protocol.

8.16 Departure from the protocol

Additional tests/procedures may be performed for clinical management at the discretion of the attending physician.

Notification that additional tests/procedures are being performed or of desired protocol amendments will be sent to the responsible IRB(s), Clinical Monitors, Study Coordinator, USAMMDA. In addition, departures from the approved protocol will be noted and reported to the IRBs in a timely manner.

Changes to the approved protocol will be approved by the Tunisian IRB and by the Human Subjects Research Review Board (HSRRB) prior to implementation. Changes intended to eliminate an apparent immediate hazard to a subject will be immediately implemented provided the reviewing IRBs are notified.

Any departures from the protocol that significantly affect the integrity of the study of the safety and welfare of subjects will be promptly reported to the HSRRB.

8.17 Procedural timetable

Procedure	Therapy Period (days)		Follow-up (#Days)	
	0/1	10	20	50±7	90±7
Informed Consent	X				
Demographic Details	X				
Medical History	X				
History of Leishmaniasis	X				
Physical Exam	X				
Dermatology Exam	X				
Hearing Test	X	X	X		
Romberg Test	X	X	X		
CBC (WBC,platelets, Hgb)	X				
AST or ALT	X				
Serum Creatinine	X	X	X		
Glucose, Na, K	X				
Pregnancy Test	X				
Urinalysis	X		X		
Measure ulcer and induration	X	X	X	X	X
Clinical Evaluation of Lesion	X	X	X	X	X
Photograph Lesion	X	X	X	X	X
Parasitologic Tests	X				
Cutaneous Biopsy	X	X			
Aminoglycoside concentration	X	X			
Parasite load	X	X			
Leishmania Culture (Microdilution Test)	X	X			
Parasite load by DNA and RNA techniques	X	X			
Entrance Exam and Lab Test Check List	X				

Drug Therapy	Days 1 through 20
Local Toxicity	Days 1 through 20

9.0 SAMPLE SIZE AND DATA ANALYSIS

9.1 Sample size calculation

If the cure rate in the WR 279,396 without occlusion group is assumed to be 60%[1], and we desire at least a 35% improvement in the cure rate due to occlusion, for alpha=0.05 (1-sided) and beta=0.2, 20 patients under WR 279,396 without occlusion plus 20 patients WR 279,396 with occlusion (40 patients) are required for 1:1 allocation.

9.2 Efficacy calculations

Data analysis will be undertaken in 3 stages: conception of an electronic database from the questionnaire, data entry and cleaning to insure validity of data and statistical analysis: A database will be written from the CRF. Data entry with a double-check will be performed at the end of the protocol at the Tunisian site with U.S. support. Cleaning of the data base will be insured by comparing data entered to the computer to the information in the source documents. Data analysis will be done by an independent qualified statistician hired in the United States for this phase of the protocol.

The primary criterion for treatment evaluation will be the percentage of patients that demonstrate complete clinical response (all lesions cured or improved) at day 50. The response rate for WR 279,396 groups will be compared by Fisher's Exact test. Time for the ulcer to cure will also be determined and the means will be used to compare the two groups. Results will also be analyzed per cohort (Tegaderm VS no Tegaderm) and the age of the lesion at treatment D0 (≤ 2.5 months of age VS ≥2.5 months of age).

The secondary efficacy calculation will be the deep lesional dermis parasite load log reduction ratio at Day 10 of treatment with/without occlusion. Mean parasite loads in the two groups, will be compared using student's t tests.

As for all efficacy calculations the data analysis for safety and tolerance of WR 279,396 will be done by a qualified statistician and results between the two groups compared.

9.3 Treatment randomization codes

The study will be randomized 1:1 occlusion/non occlusion. The randomization list will be generated by the Department of Epidemiology of Institut Pasteur of Tunis using a sequential numbers from 1 to 50.

[1] In Colombia, WR279396 without occlusion gave a 60% cure rate (30% above the placebo cure rate).

9.4 Missing or unused data

The Case Report Form was designed to capture the same result multiple times making it very hard to have missing data. In addition, check lists are common throughout the protocol. Nevertheless, if data is missing the PIs will try their best to obtain the missing data. Unused data will not be published. If a subject is lost to follow-up prior to day 50, the data will not be included in the analysis. If a subject is lost to the study between day 50 and the third month follow-up, the PIs will have the option to use the data with remarks.

10.0 DIRECT ACCESS TO SOURCE DATA/DOCUMENTS

The investigators, medical monitors, study monitor, study coordinator members of USAMMDA, members of the Institut Pasteur in Tunisia, Tunisian Minister of Health, and other government agencies Tunisian, and the U.S. Army Surgeon General’s Human Subjects research Review Board as part of their duties, may access study data to monitor, audit, IRB review and pursue regulatory inspections of this trial's source data and documents as part of their responsibility to protect human subjects in research.

11.0 QUALITY CONTROL AND QUALITY ASSURANCE

USAMMDA will provide in-country quality control and quality assurance in accordance with good clinical practices.

12.0 ETHICS

The risks to normal volunteers associated with participation in this study are relatively low, whereas the potential benefit to society in the successful development of an effective topical treatment for cutaneous leishmaniasis skin is high. Therefore, on balance, the study stands on solid ethical basis.

The study will be explained to the subject/Legal Representative (LR) and to minors in a language that they can understand in order to assure that they are voluntarily cooperative and willing to participate in the protocol. Once the diagnosis of Cutaneous Leishmaniasis has been made (that is parasitologically proven to be Cutaneous Leishmaniasis), the investigator will obtain the subject’s consent. The investigator will respond to all of the subject’s questions and will continue to provide information after obtaining the subject’s voluntary agreement. For the minors (≥15 years old but <18 years old), in addition to the LR-Consent form, an Assent (outlines the study in simplified language) will also be obtained. Any significant information that may affect the volunteer’s health or willingness to continue participation that is found during the study will be made available to the volunteer/LR. Volunteers will be withdrawn from this research in the event of: (1)

the development of health conditions that would make the volunteer’s continued participation in this study dangerous; or (2) the development of any other conditions which might occur that render the continued participation detrimental to the volunteer’s health.

The conduct of this trial will follow the Helsinki Accords, FDA Good Clinical Practices, U.S. Army Surgeon General regulations concerning clinical trials and Tunisian Ministry of Health policies and regulations regarding clinical trials. Progress on this protocol will be provided to the Human Use Review Committees, the Study Monitors, the study coordinator, and USAMMDA annually and as appropriate during the study.

13.0 DATA HANDLING AND RECORDKEEPING

A copy of the protocol, CRF(s), other source documents and regulatory records will be kept on file by the U.S. Army Medical Research and Materiel Command for 2 years after the marketing application has been approved or if no application is to be filed or if the application was not approved for such indication for 2 years after the investigation was discontinued and the FDA is notified. As a co-sponsor, and according to Tunisian laws, copies of the protocol, CRF(s), other source documents and regulatory records will be kept by the Institut Pasteur in Tunis for 2 years after the marketing application has been approved or if no application is to be filed or for 15 years after the investigation is discontinued.

14.0 FINANCING

The financing of this trial is to come from the Tunisian Ministry of Health, Institut Pasteur Paris and U.S. Army Medical Materiel Development Activity (USAMMDA)

15.0 PUBLICATION POLICY

If journal articles are to be written then no volunteer will have their name or initials released, they will be identified by their volunteer number. Some of the photos may be used. It is anticipated that the results of this study will be presented to the scientific community via oral presentations at meetings and written publications in scientific journals.

16.0 VOLUNTEER REGISTRY DATA BASE REQUIREMENTS

It is the policy of the U. S. Army Medical Research and Materiel Command that data sheets (USAMRDC Form 60-R) are to be completed on all volunteers participating in research for entry into this Command's Volunteer Registry Database. The information to

be entered into this Subject confidential database includes your name, address, study number, study name, and dates. The intent of the database is two-fold: first, to readily answer questions concerning an individual's participation in research sponsored by USAMRMC; and second, to ensure that USAMRMC can exercise its obligation to ensure research volunteers are adequately warned (duty to warn) of risks and to provide new information as it becomes available. The information will be stored at USAMRMC for a minimum of 75 years. For The U.S. Army Medical Research and Materiel Command will go through the Institute Pasteur in Tunis to contact the subject if necessary, as the Tunisian law prohibits the release of Social Security Numbers.

17.0 ASSESSMENT OF SAFETY

17.1 Safety Parameters / Adverse Events

The recording of adverse events is the responsibility of the investigators. Volunteers will be instructed to contact the investigators immediately in the event they develop any unusual signs or symptoms post treatment. Follow-up information will be provided for all initial reports of serious and unexpected adverse events as described in 16.2, below.

17.1.1 Adverse Event

An adverse event temporally related to participation in the study should be documented, whether or not it is considered to be related to the test article. This definition includes intercurrent illnesses and injuries, or exacerbation of preexisting conditions. The following will be included in all IND safety reports: Subject identification number and initials; investigator’s name and name of MTF; subject’s date of birth, gender, and ethnicity; test article and dates of administration; signs/symptoms and severity; date of onset; date of resolution or death; relationship to the study drug; action taken; concomitant medication(s), including dose, route and duration of treatment, date of last dose.

17.1.2 Serious Adverse Event

A serious adverse event is any untoward medical occurrence that at any dose:

* results in death
* is life-threatening [Life threatening event is an event which presents the risk of death at the time of the event; it does not refer to an event which hypothetically might have caused death had it been more severe.]
* requires inpatient hospitalization
* results in persistent or significant disability/incapacity [Disabling/Incapacitating adverse event is any event, which may result in a substantial disruption of the volunteer’s ability to carry out normal life functions. This definition is not intended to include minor cases of

headache, nausea, vomiting, diarrhea, influenza, rhinorrhea, lacrimation or accidental trauma, such as a sprained ankle.]

- Important medical events that may not result in death, may not be life-threatening or require hospitalization may be considered serious when, based on the medical judgement of the investigator, they may adversely affect the subject and may require medical/surgical intervention (i.e. an allergic reaction resulting in bronchospasm requiring immediate intervention without requiring hospitalization).

17.2 Recording and Reporting Adverse Events

17.2.1 Documenting Adverse Events

At the time of each visit all adverse events either observed or reported, will be documented in the CRF and in the subject's medical records. The investigator and clinical monitor team will evaluate each adverse event. In the event a medical diagnosis is made, the event will be reported as an AE or an SAE. If no medical diagnosis is made, individual data will be documented as the AE/SAE in the CRF. Details of any therapeutic measures taken in the event of AE/SAE will be recorded. Adverse events previously documented in the CRF will be recorded as 'ongoing', 'improved' or 'resolved' at subsequent visits. If an adverse event changes, or advances in quantity or quality, a new record of the event will be initiated.

17.2.2 Reporting Adverse Events

Reporting of serious and unexpected adverse events: All serious adverse events and all unexpected adverse events will be immediately reported to the Pharmacovigilance service of the Institut Pasteur of Tunis The information will be sent by facsimile to 216 71 781 933. A written report will follow within 3 working days. In addition, serious and unexpected adverse experiences will be immediately reported by telephone to the Clinical Monitor Dr. Nissaf Ben Alaya to Tel: 216 71 792 429, and the study Principal Investigator Dr. Afif Ben Salah to 216 71 792 429 and the Study Coordinator Dr. Max Grogl 808-433-7171. The US Army MRMC Deputy for the Office of Research Protections will be also informed at 011-301-619-7802. Written follow-up information and the medical monitor's written review and assessment of the adverse event will be provided to the HSRRB and USAMMDA.

Unanticipated problems involving risk to subjects or others, serious adverse events related to participation in the study and all subject deaths should be promptly reported by phone (301-619-2165), by email (hsrrb@amedd.army.mil), or by facsimile (301-619-7803) to the Army Surgeon General's Human Subjects Research Review Board (HSRRB). A complete written report should follow the initial notification. In addition to the methods above, the complete report can be sent to the U.S. Army Medical Research and Materiel Command, ATTN: MCMR-ZB-P, 504 Scott Street, Fort Detrick, Maryland 21702-5012."

Routine reporting: will apply to local and non-serious systemic events.

17.2.3 Follow-Up of Adverse Events

The investigator will determine causality of the AE/SAE. This may include additional laboratory testing, follow up visits and/or histopathological examinations. All serious adverse events will be followed until resolution or SAE becomes stable. Non-serious adverse events will be followed until study conclusion. Reports relative to the subsequent course of an adverse event noted for any volunteer will be submitted to the local Medical Monitors.

17.3 Study Specific Definitions and Exceptions

Any hospitalization following initiation of the study for an elective procedure or therapy will be reported as a 'hospitalization (Not adverse event)' on the CRF. If this hospitalization is planned prior to study participation or arises from a pre-existing condition, it will be recorded on the medical history form and the CRF. For study specific definitions side effects and grades see Appendix A.

18.0 ROLES AND RESPONSIBILITIES OF THE STUDY PERSONEL

Principal Investigators: Responsible for protocol adherence and execution, passage of protocol through Tunisian protocol committee, tie-breaking blinded assessment of lesion changes in patients, data integrity, applying for and receiving approval for any modifications to the protocol or consent form in Tunisia, training of associate investigators, ensuring safety of the volunteers, and reporting of any adverse events.

Subinvestigators: Responsible for protocol adherence and execution, passage of protocol through Tunisian protocol committee, tie-breaking blinded assessment of lesion changes in patients, data integrity, applying for and receiving approval for any modifications to the protocol or consent form, ensuring safety of the volunteers, managing and reporting of any adverse events, briefing potential volunteers, obtaining proper informed consent, determining study eligibility based on screening data and the exclusion criteria, and recording all observations and data in the individual subject records.

Medical Monitors: Responsible to provide medical care and monitoring of research subjects for conditions that may arise during the conduct of the study. The medical monitor will review all serious adverse events and all unexpected adverse events (per ICH definitions) associated with the protocol and provide an unbiased written report of the event within 10 calendar days of the initial report. At a minimum, the medical monitor will comment on the outcomes of the adverse event (AE) and relationship of the AE to the test

article. The medical monitor will also indicate whether he/she concurs with the details of the report provided by the study investigator.

Coordinator Consultant and Study Monitor: Assures overall coordination of the study and protocol writing in collaboration with the PIs. Assures the study is conducted under cGCP in collaboration with the QC unit at USAMMDA. Uses his expertise in the product and previous experiences in Phase 1 and Phase 2 studies with WR 279,396 in support the Principal Investigators. Reviews weekly Reports and Summary Study Reports and communicates with the PIs accordingly. As a member of the Product Development Team keeps lines of communication open with the Product Managers and update other members of the Product Development Team in the United States by phone. Reviews and updates for the product managers the Annual Reports and Final reports.

19.0 REFERENCES

1. Berman JD. 1988. Leishmaniasis Chemotherapy: biochemical mechanisms, clinical efficacy, and future strategies. Rev Infect Dis 10:560-586.

2. Soto- Mancipe J, Grogl M, Berman JD. 1993. Evaluation of pentamidine for the treatment of cutaneous leishmaniasis in Colombia. Clin Infect Dis 16: 417-425.

3. Asilian A, Jalayer T. 1995. A randomized placebo-controlled trial of a two-week regimen of aminosidine (paromomycin) ointment for treatment of cutaneous leishmaniasis in Iran. Am J Trop Med Hyg 53(6): 648-651.

4. Ben Salah A, Zakraoui H. 1995. A randomized placebo-controlled trial in Tunisia treating cutaneous leishmaniasis with paromomycin ointment. Am J Trop Med Hyg 53(2): 162-166.

5. Soto J, Grogl M, Berman J, Olliaro P. 1994. Modest efficacy of injectable aminosidine for single-agent therapy for Colombian cutaneous leishmaniasis. Trans Roy Soc Trop Med Hyg 88: 695-698.

6. Harms G, Chehade AK. 1991. A randomized trial comparing a pentavalent antimonial drug and recombinant interferon-gamma in the local treatment of cutaneous leishmaniasis. Trans R Soc Trop Med Hyg 85(2): 214-216.

7. Bellazoug S, Neal RA. 1986. Failure of meglumine antimoniate to cure lesions due to L. major in Algeria. Trans R Soc Trop Med Hyg 80(4): 670-671.

8. Herwaldt BL, Berman JD. 1992. Recommendations for treating leishmaniasis with sodium stibogluconate (Pentostam) and review of pertinent clinical studies. Am J Trop Med Hyg 46: 296-306.

9. Arana BA, Navin TR, Arana FE, Berman JD, Rosenkaimer F. 1994. Efficacy of short-course (10 days), high-dose meglumine antimonate with or without interferon-gamma in treating cutaneous leishmaniasis in Guatemala. Clin Infect Dis 18:381-384.

10. Jeannel D, Tupin P. 1989. Leishmaniasis in France. Lancet 8666: 804.

11. Caumes E, Carriere J. 1995. Dermatosis associated with travel to tropical countries: a prospective study of the diagnosis and management of 269 patients presenting to a Tropical Disease Unit. Clin Infect Dis 20(3):542-548.

12. Sharifi I, Fekri R. 1998. Bull World Health Organ 76(3): 289-293.

13. El-On J, Livshin R, Evan-Paz Z, Hamburger D, Weinrauch L. 1986. Topical treatment of cutaneous leishmaniasis. J Invest Dermatol 87:284-8.

14. El-On J, Greenblatt CL. An in vitro model for testing the effect of anti-leishmanial drugs of
possible use in topical treatment. 1983. Current Therapeutic Research 33:660-669.

15. El-On J, Messer G. Leishmania major: antileishmanial activity of methylbenzethonium chloride. 1986. Am J Trop Med Hyg 35: 1110-1116.

16. Soto J, Hernandez N, Mejia H, Grogl M, Berman J. 1995. Successful treatment of American CL with a combination of topical paromomycin/MBCl and injectable Glucantime. Clin Infect Dis 20: 47-51.

17. Glaza SM. 1995. Dermal sensitization study of WR 279396, an antileishmanial topical cream in Guinea Pigs-closed patch technique. HWI #40501111.

18. Glaza SM. Primary dermal photo activated irritation study of WR 279396, an antileishmanial topical cream in rabbits. HWI # 40501110.

19. Glaza SM. 1995. Photoallergic contact dermatitis study of WR 279296, an antileishmanial topical cream in Guinea Pigs (Armstrong Method). HWI # 40501112.

20. Levine BS. 1995. Four Week dermal toxicity of study of WR 279396 in CD Rats. [Verbal communication from Dr. Levine; written confirmation to be sent 23 June 95.]

21. Aminosidine [paromomycin] Product Profile. Undated. Farmitalia Carlo Erba. Milan, Italy.

22. Wilfert JN, Burke JP, Bloomer HA, Smith CB. 1971.Renal insufficiency associated with gentamicin therapy. J Infect Dis 124: S148-S153.

23. Anonymous. 1993. Humatin. Physicians Desk Reference: 1776.

24. El-On J, Halevy S, Grunwald MH, Weinrauch L. 1992. Topical treatment of Old World cutaneous leishmaniasis caused by Leishmania major: a double blind control study. J Amer Acad Dermatol 27:227-31.

25. El-Safi SH, Murphy AG, Bryceson ADM, Neal RA. 1990. A double-blind clinical trial of the treatment of cutaneous leishmaniasis with paromomycin ointment. Trans R Soc Trop Med Hyg 84: 690-691.

26. Anonymous. 1993. Garamycin. Physicians Desk Reference: 2187-2190.

27. Sande MA, Mandell GL. 1983. The Aminoglycosides. in Gilman AG, Goodman LS, Rall TW, Murad F Eds. The Pharmacological basis of Therapeutics. 7th Edition. McMillan Publ Co. NY. Chapter 51.

28. Jackson GG, Arcieri G. 1971. Ototoxicity of gentamicin in man: a survey and controlled analysis of clinical experience in the United States. J Infect Dis 124: S130-S137.

29. Stone HH, Kolb LD, Pettit J, Smith RB. 1968. The systemic absorption of an antibiotic from the burn wound surface. American Surgeon 34: 639-643.

30. Dayal VA, Smith EL, McCain G. 1974. Cochlear and vestibular gentamicin toxicity. Arch Otolaryngol 100: 338-340.

31. Parnes LS, Riddell, D. 1993. Irritative spontaneous nystagmus following intratympanic gentamicin for Meniere's disease. Laryntoscole 103 745-749

32. Campochiaro PA, Lim JI. 1994. Aminoglycoside toxicity in the treatment of endophthalmitis. Arch Ophthalmolo 112: 48-53.

33. Bonner M. 1996. Irritant and phototoxicity reactions to the topical antileishmanial WR 279396: a randomized, double-blind phase I study. WRAIR.

34. Franke E, et al. 1990. Efficacy and toxicity of sodium stibogluconate for mucosal leishmaniasis. Ann Int Med 113: 934-940.

35. Alrajhi A, et al. 2002. Fluconazole for the treatment of cutaneous leishmaniasis caused by Leishmania major. N Engl J Med 346(12): 891895.

20.0 SIGNATURE OF INVESTIGATORS AND MEDICAL MONITOR

I, the undersigned, have reviewed this protocol, including Appendices, and I will conduct the study as describe and will adhere to the Ethical and Regulatory Considerations delineated herein. I was informed on the principles and requirements of Good Clinical Practices.

20.1 PRINCIPAL INVESTIGATOR:

_______________________________________ Date: ____________________
Afif Ben Salah, MD

20.2 SUBINVESTIGATORS:

_______________________________________ Date: ____________________
Nathalie Messaoud, MD

20.3 SCIENTIFIC EXPERT AND CONSULTANT

_______________________________________ Date: ____________________
Pierre Buffet, MD, Ph.D.

20.4 MEDICAL MONITOR:

_______________________________________ Date: ____________________
Dr. Nissaf Ben Alaya, MD

20.5 STUDY COORDINATOR AND CONSULTANT:

_______________________________________ Date: ____________________
Dr. Max Grogl, Ph.D.

Appendix A (Adverse or Unexpected Event Determination – Side Effects and Grades)

1. Cutaneous adverse reactions (i) on the edge of lesion (ii) on surrounding healthy skin [grade erythema separately from edema]

Grade 0: no reaction (no erythema or edema)
Grade 1: mild reaction (barely perceptible erythema or edema)
Grade 2: moderate reaction (well defined erythema or edema)
Grade 3: Severe reaction (very red erythema with raised > 2mm edema)
Grade 4: life threatening reaction (exfoliative dermatitis)

NOTE: Grade 3 reactions require immediate notification to Medical Monitor.
Grade 4 reactions require immediate termination of treatment and ADVERSE EVENT NOTIFICATION.

2. Abnormal clinical laboratory values (DAIDS/NIH scale):

Parameter (units)	Grade 1	Grade 2	Grade 3	Grade 4
Creatinine (xULN)	>1-1.5	>1.5-3	>3-6	>6
AST/ALT (XULN)	1.25-2.5	>2.5-5	>5-10	>10
WBC (1,000/mm3)	1000-1500	750-999	500-749	<500
PLT (1,000/mm3)	75-99	50-74	20-49	<20
HGB (g/dL)	8-9.4	7-7.9	6.5-6.9	<6.5

NOTE: Grade 2 require immediate notification to Medical Monitor.
Grade 3 and 4 reactions require immediate termination of treatment.
Grade 2, 3 and 4 laboratory values require evaluation of the volunteer. If the laboratory abnormality reflects clinically significant pathology, an ADVERSE EVENT report will be fill out and notification will occur.

3. Miscellaneous (associated with drug administration):

Pain:
Grade 0: none (feels no pain)
Grade 1: mild pain (does not interfere with daily activity)
Grade 2: moderate pain (interferes with daily activity)
Grade 3: severe pain (daily activities are interrupted)

NOTE: Grade 2 reactions require immediate notification to Medical Monitor.
Grade 3 reactions require immediate termination of treatment and ADVERSE EVENT NOTIFICATION.

Vertigo:
Grade 0: none (feels no vertigo)
Grade 1: mild vertigo (does not interfere with daily activities)
Grade 2: moderate vertigo (interferes with daily activity)
Grade 3: severe vertigo (daily activities are interrupted)

NOTE: Grade 2 reactions require immediate notification to Medical Monitor.
Grade 3 reactions require immediate termination of treatment and ADVERSE EVENT NOTIFICATION.

Tinnitus:
Grade 0: none (feels no ringing/buzzing/roaring etc.)
Grade 1: mild tinnitus (does not interfere with daily activities)
Grade 2: moderate tinnitus (interferes with daily activities)
Grade 3: severe tinnitus (daily activities are interrupted)

NOTE: Grade 2 reactions require immediate notification to Medical Monitor.
Grade 3 reactions require immediate termination of treatment and ADVERSE EVENT NOTIFICATION.

Diminished hearing:
Grade 0: none (stays within normal limits) Normal = -10 db to 25 db
Grade 1: mild hearing loss (26 db - 40 db)
Grade 2: moderate hearing loss (41 db - 55 db)
Grade 3: severe hearing loss (56 db - 80 db)
Grade 4: profound hearing loss (> 80 db)

NOTE: Grade 2 reactions require immediate notification to Medical Monitor.
Grade 3 and 4 reactions require immediate termination of treatment and ADVERSE EVENT NOTIFICATION.

4. Reporting of adverse events:

Serious and unexpected adverse experiences will be immediately reported to the Pharmacovigilance service of the Institut Pasteur of Tunis The information will be sent by facsimile to 216 71 781 933. A written report will follow within 3 working days. In addition, serious and unexpected adverse experiences will be immediately reported by telephone to the Clinical Monitor Dr.Nissaf Ben Alaya to Tel: 216 71 792 429, and the study Principal Investigator Dr. Afif Ben Salah to 216 71 792 429. The US Army MRMC Deputy for the Office of Research Protections will be also informed at 011-301-619-7802. Written follow-up information and the medical monitor's written review and assessment of the adverse event will be provided to the HSRRB and USAMMDA.
